# Diagnosis and Management of Acute Coronary Syndrome: What is New and Why? Insight From the 2020 European Society of Cardiology Guidelines

**DOI:** 10.3390/jcm9113474

**Published:** 2020-10-28

**Authors:** Paul Guedeney, Jean-Philippe Collet

**Affiliations:** Institut de Cardiologie, Sorbonne Université, ACTION Study Group, INSERM UMRS_1166, Hôpital Pitié-Salpêtrière (Assistance Publique-Hôpitaux de Paris), 75013 Paris, France; pguedeney@hotmail.fr

**Keywords:** acute coronary syndrome, oral anticoagulation, antiplatelet, percutaneous coronary intervention

## Abstract

The management of acute coronary syndrome (ACS) has been at the center of an impressive amount of research leading to a significant improvement in outcomes over the last 50 years. The 2020 European Society of Cardiology (ESC) Guidelines for the management of patients presenting without persistent ST-segment elevation myocardial infarction have incorporated the most recent breakthroughs and updates from large randomized controlled trials (RCT) on the diagnosis and management of this disease. The purpose of the present review is to describe the main novelties and the rationale behind these recommendations. Hence, we describe the accumulating evidence against P2Y_12_ receptors inhibitors pretreatment prior to coronary angiography, the preference for prasugrel as leading P2Y_12_ inhibitors in the setting of ACS, and the numerous available antithrombotic regimens based on various durations of dual or triple antithrombotic therapy, according to the patient ischemic and bleeding risk profiles. We also detail the recently implemented 0 h/1 h and 0 h/2 h rule in, rule out algorithms and the growing role of computed coronary tomography angiography to rule out ACS in patients at low-to-moderate risk.

## 1. Introduction

Despite tremendous achievements in its management, coronary artery disease (CAD) remains a leading cause of mortality worldwide [1,2]. Acute coronary syndrome, the most severe manifestation of CAD, is burdened by a significant mortality, concerning approximately 5%–8% of the cases within six months of diagnosis [3]. To further improve outcomes following acute coronary syndrome (ACS), it is paramount for physicians dealing with such patients to implement in clinical practice the latest findings from large RCTs. The purpose of the ESC guidelines is to summarize and evaluate available evidence to facilitate decision making processes and to propose the best management of patients according to their specific situations and potential comorbidities. This year, the ESC updated their guidelines with respect to the diagnosis and management of patients presenting with non-ST-segment-elevation ACS (NSTE-ACS) [4,5]. The purpose of the present review is to summarize the main novelties of these guidelines and detailed the evidence and data that led to these updates.

## 2. Diagnosis of Acute Coronary Syndrome 

### 2.1. Rule-In, Rule-Out Algorithms

**Figure 1:**
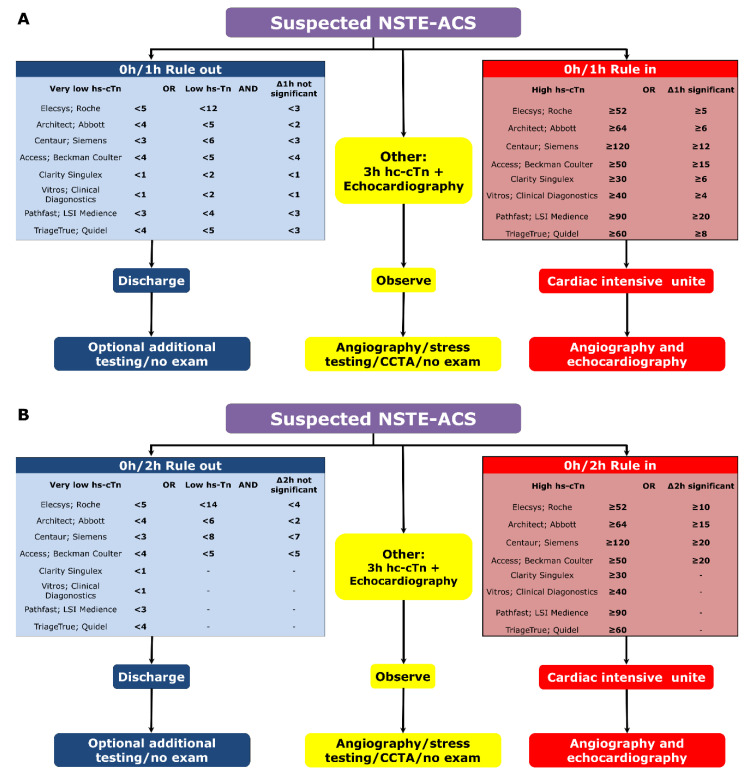
Rule in/Rule out algorithms according to the 2020 ESC NSTEMI guidelines: 0 h/1 h algorithm (**A**) and 0 h/2 h algorithm (**B**).STE-ACS: non-ST-segment elevation myocardial infarction; cTn: cardiac troponin; CCTA: coronary computed tomography angiography.

Measurement of cardiac troponin (cTn) T or I is mandatory for the diagnosis and risk stratification of an ACS. Over the last decade, use of high-sensitivity (Hs) assays has considerably grown in clinical practice, allowing for more rapid detection of troponin elevation, within one hour of symptom onset, and with improved sensibility and specificity [5]. Based on the rational that Hs-cTn is a continuous variable with early (i.e., within 1 h or 2 h) absolute changes being surrogate of later (within 3 h or 6 h) absolute changes, the 2015 ESC guidelines recommended to use rapid rule-out and rule-in protocols. The ESC 0 h/1 h algorithm is based on a blood sample at 0 h and 1 h thereafter, using validated thresholds for both baseline and variation (i.e., Δhs-cTn) levels of hs-cTn (Figure 1A,B).

The 2020 ESC Non-ST-segment elevation Myocardial infarction (NSTEMI) guidelines have extended these recommendations to include validated 0 h/2 h algorithms, following recent publications (Figure 1B) [6,7,8]. Conversely, the more historical ESC 0 h/3 h algorithm was demoted from a class I recommendation to a class IIa, following the results of 3 larges diagnosis studies which suggested that the ESC 0 h/1 h algorithms were associated with improved safety and efficacy [9,10,11]. In case of ruled-out-patients, or patients for whom electrocardiogram or hs-cTn dosage may be inconclusive, coronary computed tomography angiogram (CCTA) may be readily performed as an alternative to invasive coronary angiography to exclude ACS (class I recommendations, level A of evidence) [5]. In a recent study, 207 patients presenting with acute chest pain, elevated hs-cTn and inconclusive electrocardiogram were randomized to a strategy cardiovascular magnetic resonance imaging or CCTA-first versus standard of care [12]. While all patients randomized to the standard of care underwent coronary angiogram, only 67% of the patient randomized to CCTA-first underwent such exam (*p* < 0.001), without significant difference in term of major adverse cardiac events at one year. Moreover, a subanalysis of the very Early Versus Deferred Invasive Evaluation Using Computerized Tomography in Patients with Acute Coronary Syndromes (VERDICT) trial has confirmed the very high negative predictive value of CCTA [13]. In this study, CCTA was performed prior to coronary angiogram in 1023 patients and was associated with negative predictive value of 90.9%, 95% confidence interval (CI) 86.8–94.1%, with a sensitivity and specificity of 96.5%, 95% CI 94.9–97.8%, and 72.4%, 95% CI 67.2–77.1%, respectively.

### 2.2. Other Biomarkers

B-type natriuretic peptide (BNP) or N-terminal pro-BNP (NT-proBNP) may be useful for the diagnosis and evaluation of the severity of heart failure in the setting of ACS [14,15]. Systematic assessment of other biomarkers, such as C-reactive protein or copeptin, though associated with outcomes following ACS, is no longer recommended as their prognosis value compared to BNP/NT-proBNP or the Global registry of Acute Coronary Events (GRACE) score remains limited [16,17,18].

## 3. Management of The Antithrombotic Treatment

### 3.1. The Issue of Pretreatment

Pretreatment refers to the administration of aspirin and P2Y_12_ receptor inhibitors prior to the coronary angiogram [19]. The rationale behind pretreatment is ensuring an adequate platelet inhibition as fast as possible once the diagnosis of ACS is suspected. Pharmacological studies have indeed demonstrated that several hours may be necessary for the achievement of sufficient platelet inhibition following oral administration of a loading dose, even when using potent P2Y_12_ inhibitors such as prasugrel or ticagrelor [20]. Notwithstanding, pretreatment may expose patients to an unnecessary risk of bleeding if the diagnosis of ACS is eventually disproved following coronary angiogram, which may be the case in up to 35% of patients [21]. Until recently, large RCT evaluating the safety and efficacy of pretreatment in the setting of ACS have been scarce. The largest RCT on this topic has been the Comparison of prasugrel at the Time of Percutaneous Coronary Intervention (PCI) or as Pretreatment at the Time of Diagnosis in Patients with Non-ST Elevation Myocardial Infarction (ACCOAST) trial which compared a strategy of systematic prasugrel pretreatment with a 30 mg loading dose followed by an additional 30 mg of prasugrel in case of PCI to a strategy of 60 mg loading dose of prasugrel only in case of PCI [21] in NSTEMI patients. In this trial, the pretreatment strategy did not impact the rate of the composite endpoint of death from cardiovascular causes, myocardial infarction (MI), stroke, urgent revascularization or glycoprotein IIb/IIIa inhibitor bailout within 7 days (Hazard ratio[HR] 1.02, 95%CI 0.84–1.25, *p* = 0.81, but it significantly increased the risk of Thrombolysis in Myocardial Infarction (TIMI) life-threatening and major bleeding not related to coronary artery bypass grafting (CABG) (HR 5.56 95%CI 1.63–19.0 and HR 2.95, 95%CI 1.39–6.28, respectively). These findings were further confirmed in the PCI-only cohort and were not impacted by the timing of angiography, when performed within the first 48 h of randomization [22,23]. Following the ACCOAST trial, the ESC guidelines have recommended against pretreatment, but only with prasugrel, and without specific recommendations for pretreatment with ticagrelor. This distinction was likely the consequence of the Platelet Inhibition and Patient Outcomes (PLATO) trial, which included patients presenting with ACS and compared ticagrelor to clopidogrel, which were both administered following randomization and prior to coronary angiogram [24]. In a post-hoc analysis of the PLATO trial, only including patients presenting with NSTEMI, treatment with ticagrelor was associated with a reduction of cardiovascular death, MI, or stroke and all-cause death, with no significant increase of major bleeding within the first 10 days of randomization, and regardless of whether patients underwent revascularization or not [25]. It is important to note however, that pretreatment was applied to both ticagrelor and clopidogrel and was not the subject of randomization.

**Figure 2:**
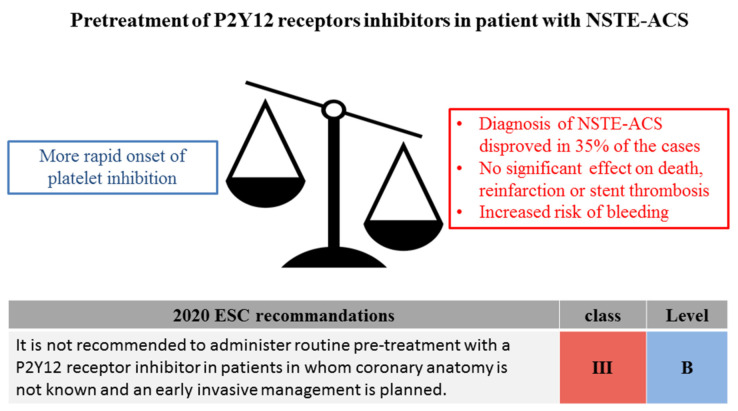
Pretreatment with P2Y_12_ receptors inhibitors in patients presenting with non-ST-segment elevation myocardial infarction. NSTE-ACS: non-ST-segment elevation myocardial infarction; ESC: European society of Cardiology.

The debate on this topic has nonetheless been settled with the recent publication of several trials directly evaluating the safety and efficacy of pretreatment. First, the Rapid Early Action for Coronary Treatment (ISAR-REACT) 5 was an open-labeled trial which included 4018 patients presenting with an ACS and scheduled to undergo coronary angiography [26]. The trial compared a strategy of systematic pretreatment with ticagrelor to a strategy with prasugrel where the loading dose was only administered in case of PCI unless patients were presenting with STEMI. In this trial, treatment with ticagrelor and systematic pre-treatment was associated with a significant increase of the risk of death, MI, or stroke at one year (HR 1.36 95% CI 1.09–1.70), demonstrating that a more rapid onset of platelet inhibition did not result into long term clinical benefice. These results were further confirmed by the recently published downstream versus upstream administration of P2Y_12_ receptor blockers in non-ST elevated acute coronary syndromes with initial invasive indication (DUBIUS) trial [27]. The DUBIUS trial was an open-label study where patients presenting with NSTEMI and planned to undergo coronary angiography were randomized to an upstream strategy based with ticagrelor pretreatment or downstream strategy based on either ticagrelor or prasugrel, solely administered in case of PCI. The trial was prematurely interrupted for futility after the inclusion of 1449 patients. There was no significant difference between the two strategies with respect to the composite endpoint of death due to vascular cause, MI, stroke, or Bleeding Academic Research Criteria (BARC) type 3, 4, or 5 bleeding as well as each individual endpoint. These data from randomized trials have been confirmed by large real-world registries. The ARIAM-Andalucía registry retrospectively evaluated 9621 patients presenting with ACS and managed invasively [28]. The study, stratified according to the type of ACS, reported a statistically significant benefice for pretreatment with clopidogrel in case of STEMI, with lower risk of reinfarction (Odds Ratio [OR] 0.53 95%CI 0.27–0.96), stent thrombosis (OR 0.67 95% 0.48–0.94) and mortality (OR 0.67 95%CI 0.48–0.94). Interestingly, the benefits of pretreatment were no longer present when evaluating patients presenting with NSTE-ACS. More recently, a study from the Swedish Coronary Angiography and Angioplasty Registry (SCAAR) evaluated 64,857 patients undergoing PCI for NSTEMI from 2010 to 2018, the vast majority of whom (i.e., 92.4%) were pretreated, mostly with ticagrelor [29]. In this study, pretreatment did not result in improved survival at one month (OR 1.17 95%CI 0.66–2.11) or one year (OR 1.34 95%CI 0.77–2.34), nor did it significantly reduce the risk of stent thrombosis at 30 days (OR 0.81 95%CI 0.42–1.55). However, pretreatment resulted in increased risk of in-hospital bleeding (OR 1.49% 95%CI 1.06–2.12). Following this accumulation of evidence demonstrating the lack of benefice in term of ischemic prevention with a consistent increase of the risk of bleeding complications associated with pretreatment, the 2020 ESC NSTEMI guidelines have recommended against the routine administration of P2Y_12_ inhibitors in patients in whom coronary artery anatomy is not known and an early invasive management is planned (Figure 2) [5].

However, the door is left open and “Pre-treatment with P2Y_12_ inhibitors may be considered (class IIb) in patients with NSTEMI who are not planned to undergo an early invasive strategy and are not at high risk of bleeding”.

### 3.2. What P2Y_12_ Receptors Inhibitors Should Be Used?

A personalized approach has been chosen in these guidelines to offer all possibilities according to the patient risk. When the bleeding risk is very high (i.e., planned surgery and presence of one major criteria of the Academic Research Consortium for High Bleeding Risk [ARC-HBR] or PRECISE-DAPT score ≥ 25) or high (one major criteria of the ARC-HBR or PRECISE-DAPT score ≥ 25), clopidogrel should be the preferred choice with a shorter dual antiplatelet therapy (DAPT) duration of one and three months, respectively. 

When the bleeding risk is qualified as standard, the choice must be made between ticagrelor and prasugrel. Since the landmark PLATO and therapeutic outcomes by optimizing platelet inhibition with Prasugrel-thrombolysis in myocardial infarction 38 (TRITON-TIMI 38) trials, use of DAPT based on the association of aspirin with a potent P2Y_12_ receptors inhibitors (i.e., ticagrelor or prasugrel) has been the cornerstone of the antithrombotic treatment for patients presenting with ACS [24,30]. Whether or not one of these two agents should be preferably used in the setting of ACS has remained an open debate until recently. In the ISAR-REACT 5 trial, treatment with prasugrel compared to ticagrelor was associated with a significant reduction of the primary endpoint, mainly driven by the reduction of the risk of reinfarction (HR 0.61 95% CI 0.44–0.85), without any significant difference in term of BARC type 3, 4, or 5 bleeding (HR 0.89 95% CI 0.66–1.20) [26]. In this trial, a reduced maintenance dose of 5 mg daily was used in patients aged over 75 years of with body weight <60 kg. In the dedicated substudy, which included 27.4% of the overall population, this individualized regimen of prasugrel was associated with maintained anti-ischemic efficacy compared to the one-size-fit-all ticagrelor-based strategy, while protecting these frail patients against excess risk for bleeding [31]. The physiological explanation behind the improved performance of prasugrel over ticagrelor remained to be better understood, as both agents lead to consistent platelet inhibition [20]. A recent small randomized trial reported prasugrel to be associated with improved endothelial function, as measured by the endothelium-dependent flow-mediated dilation of the radial artery following stenting, and reduced inflammation as measured by interleukin-6 level over ticagrelor or clopidogrel [32]. Ticagrelor may also induce dyspnea which may lead to increased risk of medication discontinuation [24]. According to the 2020 ESC NSTEMI guidelines, prasugrel should be considered (class IIa) in preference to ticagrelor for patients undergoing PCI for ACS without ST segment elevation [5].

### 3.3. What Antithrombotic Regimen Following PCI?

The implementation of newer generation drug-eluting, and the generalization of potent P2Y_12_ inhibitors receptors as well as lipid-lowering medication have led to a reduction of the risk of stent thrombosis and non-stent related myocardial reinfarction following PCI [33,34,35,36,37,38]. As a results, the benefice of sustained DAPT may translate into smaller absolute ischemic event risk reduction, potentially outweighed by the associated increased risk of bleeding [39]. Based on the rational that aspirin may add only limited platelet inhibition when associated to potent P2Y_12_ inhibitors, several large RCT have evaluated the safety and efficacy or early aspirin discontinuation, after 1 to 3 months of DAPT, with potent P2Y_12_ inhibitors monotherapy prolongation [40,41,42,43,44,45,46,47].

In particular, the Ticagrelor With Aspirin or Alone in High-Risk Patients After Coronary Intervention (TWILIGHT) trial randomized 7119 patients undergoing PCI, a majority of whom (i.e., 65%) for NSTEMI or unstable angina, and presenting with at least one clinical feature of high ischemic or bleeding risk and one angiographic feature of high-risk lesion to a strategy of ticagrelor monotherapy following three months of uncomplicated DAPT or prolonged DAPT [45]. The trial found ticagrelor monotherapy to be associated with a reduction of BARC type 2, 3, or 5 bleeding (4.0% vs. 7.1% HR 0.56 95%CI 0.45–0.68), without any significant difference with respect to ischemic event. 

If the TWILIGHT regimen may not be applied, patients should be treated with a 12-month duration DAPT including prasugrel or ticagrelor especially when there are high thrombotic risk criteria. However, when facing cases of patients deemed unsuitable for potent platelet inhibition, it is also possible to consider guided de-escalation of P2Y_12_ inhibitors receptors, which corresponds to switching from a potent PY12Y inhibitors (i.e., prasugrel or ticagrelor) down to clopidogrel [48]. Such de-escalation should be guided by the results of platelet function testing, as in the Testing Responsiveness to Platelet Inhibition on Chronic Antiplatelet treatment for Acute Coronary Syndromes (TROPICAL-ACS) trial, or on the results of CYP2C19-directed genotyping, which was evaluated in the Patients Outcomes after Primary PCI (Popular Genetics) trial [49,50,51]. The POPular Genetic trial, in particular, included 2488 patients undergoing PCI for STEMI reported a reduced risk of major bleeding with the genotyping-guided strategy compared to standard DAPT with ticagrelor or prasugrel (HR 0.78 95%CI 0.61–0.98) and a number needed to treat to prevent one Platelet Inhibition and Patient Outcomes (PLATO) major or minor bleeding of 37, without any significant offset in term of ischemic events [50]. 

**Figure 3:**
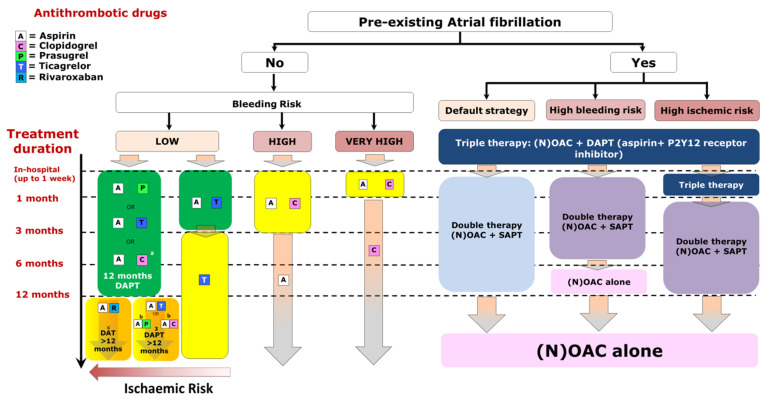
Antithrombotic regimen following PCI for NSTE-ACS according to the 2020 ESC NSTEMI guidelines. NSTE-ACS: non-ST-segment-elevation acute coronary syndrome; ESC: European society of Cardiology; (N)OAC: (non-vitamin K antagonist) oral anticoagulant; DAPT: dual antiplatelet therapy.

Extended dual antithrombotic or antiplatelet regimen may be considered in case of high-risk of ischemic event with no high risk of bleeding [52]. Several regimen may then be considered based on the results of the Prevention of Cardiovascular events in Patients With Prior heart Attack Using Ticagrelor Compared to Placebo on a Background of Aspirin-Thrombolysis in Myocardial Infarction (PEGASUS-TIMI 54), the Cardiovascular Outcomes for People Using Anticoagulant Strategies (COMPASS) and the Dual Antiplatelet Therapy (DAPT) trials (Figure 3).The main findings of these trials, along with the evaluated antithrombotic regimen are summarized in Table 1.

### 3.4. Risk Stratification

The integration of the bleeding and ischemic risks of patients undergoing PCI for an ACS is paramount as it directly impacts the type and duration of the antithrombotic regimen. The various clinical criteria previously associated with a high risk of ischemic complication following ACS are summarized in the Table 2.

According to the 2020 ESC NSTEMI guidelines, an extended long-term secondary prevention with the addition of a second antithrombotic agent (i.e., antiplatelet or anticoagulant) to aspirin should be considered in case of high thrombotic risk (class IIa) and may be considered in case of moderate thrombotic risk (class IIb) [5]. 

However, extended duration of dual antithrombotic therapy (DAT) should only be considered in the absence of high risk of bleeding. Recently, a consensus definition of high bleeding risk profile was proposed with the Academic Research Consortium for High Bleeding Risk (ARC-HBR) (Table 3) [56]. 

This pragmatic score was validated on an independent contemporary cohort, approximately half of which included patients presenting with ACS [57]. This score was introduced in the 2020 ESC NSTEMI guidelines as potential guidance tool to refine bleeding stratification, with a better sensitivity than the PRECISE-DAPT score. 

### 3.5. Pairing Chronic Oral Anticoagulation with Antiplatelet Therapy

With respect to patient requiring long-term oral anticoagulant, several large RCT including a majority of patients presenting with an ACS, evaluated DAT regimen based on non-vitamin K antagonist oral anticoagulant (NOAC) + P2Y_12_ versus triple antithrombotic therapy (TAT) with vitamin K antagonist (VKA) +aspirin+P2Y_12_ receptor inhibitors and demonstrated a significant reduction of the risk of bleeding complication with the former (Table 4) [58,59,60,61,62,63,64,65]. 

The meta-analyses of these trials have confirmed the significant reduction of bleeding and did not report any significant increase in term of ischemic event, although MI or stent thrombosis were numerically higher with DAT compare to TAT [46,66]. Consequent to this accumulation of data, the 2020 ESC NSTEMI guidelines have recommended to use a short duration of TAT (i.e., in-hospital or up to one week following PCI) in this high risk population, unless the patients also present a significant risk of ischemic risk event, and to promptly relay TAT with DAT based on the association of NOAC and P2Y_12_ inhibitors for 6–12 months according to the bleeding risk (Figure 2) [5]. Following the results of the Atrial Fibrillation and Ischemic Events with Rivaroxaban in Patients with Stable Coronary Artery Disease (AFIRE) trial, it is no longer recommended to prolonged DAT more than one year after PCI in stable patients. Indeed, the AFIRE trial randomized 2236 patients with atrial fibrillation who had undergone PCI or CABG more than one year earlier to DAT with rivaroxaban (or VKA) and a single antiplatelet agent to rivaroxaban alone [67]. The strategy of oral anticoagulant monotherapy was associated with a significant reduction of the risk of major bleeding (HR 0.59 95%CI 0.39–0.89) as well as the composite of death, stroke, MI, systemic embolism, or unstable angina (HR 0.72 95%CI 0.55–0.95).

## 4. Conclusions

The diagnosis and management of ACS remain fast-evolving fields, following the latest results from large RCTs. Antithrombotic management of ACS have known significant changes with prasugrel becoming the preferred P2Y_12_ inhibitors while pretreatment with any P2Y_12_ inhibitors is henceforth contraindicated with patients planned to undergo rapid coronary angiography. Following the acute event, antithrombotic treatment can be individualized to the ischemic and bleeding risk profiles of each patient, with numerous available regimens based on more or less prolonged triple or dual antithrombotic therapy. 

## Figures and Tables

**Table 1:** Main trials evaluating prolonged antithrombotic regimen following PCI.

Trial	Year of Publication	Main Inclusion Criteria	Proportion of Patient with ACS	Evaluated Antithrombotic Regimen	Main Results	Number Needed to Treat (Ischemic Outcomes)	Number Needed to Harm (Bleeding Outcomes)
DAPT [53]	2014	PCI followed by uncomplicated 12-month DAPT	4251/9961 (42.7%)	Prolonged DAPT with aspirin and clopidogrel (65.2%) or prasugrel (34.8%) for 18 months	Prolonged DAPT reduced ST (HR 0.29 95%CI0.17–0.48) and MACE (HR 0.71 95%CI0.59–0.85) with increased risk of moderate or severe bleeding	63	105
PEGASUS TIMI 54 [54]	2015	- Prior MI within 1 to 3 years - Age > 50 years - At least one feature among: age > 65 years; diabetes mellitus; >1 prior MI; multivessel disease, chronic kidney disease	21162/21162 (100%) including 3499/21162 (16.6%) patients with multiple prior MI	Prolonged DAPT with aspirin and ticagrelor (60 mg twice daily or 90 mg twice daily)	Both regimen of prolonged DAPT with ticagrelor reduced the risk of CV death, MI or stroke (HR 0.85 95%CI 0.75–0.96 for 90 mg b.i.d. and HR 0.84 95%CI 0.74–0.95 for 60 mg b.i.d.) and increased the risk of TIMI major bleeding (HR 2.69 95%CI 1.96–3.70 for 90 mg b.i.d. and HR 2.32 95%CI 1.68–3.21 for 60 mg b.i.d.)	84 for ticagrelor 90 mg b.i.d. 79 for ticagrelor 60 mg b.i.d.	65 for ticagrelor 90 mg b.i.d. 81 for ticagrelor 60 mg b.i.d.
COMPASS [55]	2017	- Established coronary and/or peripheral artery disease - If coronary disease and age <65 years then at least one of the following: ≥2 vascular bed disease, ≥2 risk factors among: diabetes mellitus, current smocking, chronic kidney disease, heart failure, or prior stroke	17028/27395 (62.2%) patients with prior MI	Prolonged rivaroxaban 2.5 twice daily and aspirin (100 mg once daily) or rivaroxaban (5 mg twice a day) monotherapy	DAT with rivaroxaban + aspirin was associated with a reduction of CV death, MI or stroke (HR 0.76 95%CI 0.66–0.86) but not rivaroxaban alone (HR 0.90 95%CI 0.79–1.03). Both regimens increased the risk of major bleeding (HR 1.70 95%CI 1.40–2.05 for rivaroxaban + aspirin and HR1.51 95%CI 1.25–1.84 for rivaroxaban alone)	77 for rivaroxaban + aspirin	84 for rivaroxaban + aspirin

DAPT: Dual antiplatelet therapy; ST stent thrombosis; HR: hazard ratio; CI: confidence interval; MACE: major adverse cardiac and cerebrovascular event; TIMI: Thrombolysis In Myocardial Infarction; MI: myocardial infarction; PCI: percutaneous coronary intervention. ACS: acute coronary syndrome; CV: cardiovascular.

**Table 2:** Thrombotic risk factors according to the 2020 ESC NSTEMI guidelines.

Risk Category	Complex Coronary Lesion and/or Percutaneous Procedure	High Thrombotic Risk	Moderate Thrombotic Risk
Criteria	- ≥3 stents implanted or total stent length ≥ 60 mm - ≥3 lesions treated - Critical localization: left main, last patent vessel - High-risk procedure: bifurcation, stenting with ≥2 stents or chronic total occlusion - History of stent thrombosis on antiplatelet therapy	- Complex coronary lesion and/or percutaneous procedure AND at least 1 of the following: - DM requiring medication - Recurrent MI - Polyvascular disease (CAD and peripheral disease) - Premature (<45 years) or accelerated (new lesion within 2 years of the index procedure) CAD - Concomitant systemic inflammatory disease- - CKD (eGFR between 15 and 59 mL/m^2^)	- Non-complex coronary disease or procedure AND at least 1 of the following: - DM requiring medication - Recurrent MI - Polyvascular disease (CAD or peripheral disease) - CKD (eGFR between 15 and 59 mL/min/m^2^)

CAD: coronary artery disease; ESC: European Society of cardiology; DM: diabetes mellitus; CKD: chronic kidney disease; eGFR: estimated glomerular filtration rate; MI: myocardial infarction.

**Table 3:** Academic Research Consortium for High Bleeding risk.

High Bleeding Risk: ≥ 1 Major Criterion or ≥2 Minor Criteria	
Major Criteria Chronic Oral Anticoagulation Hemoglobin < 11 g/dL Severe or End-Stage Chronic Kidney Disease Moderate or severe thrombocytopenia (<100 × 10^9^/L /L) Chronic Bleeding Diathesis Liver Cirrhosis with Portal Hypertension Active Malignancy Any History of Spontaneous Intracranial Hemorrhage or Brain Arteriovenous Malformation or Previous Traumatic Intracranial Hemorrhage Within the Past 12 Months Nondeferrable Major Surgery on Dual Antiplatelet Therapy Recent Major Surgery or Major Trauma Within 30 Days of Percutaneous Coronary Intervention	Minor Criteria Age ≥ 75 Years Moderate Chronic Kidney Disease Hemoglobin 11–12.9 g/dL for Men and 11–11.9 g/dL for Women Spontaneous Bleeding Requiring Hospitalization of Transfusion Within the Past 12 Months Long-Term Use of Oral Non-Steroidal Anti-Inflammatory History (>1 years ago) of ischemic stroke

**Table 4:** Main trials evaluating dual and triple antithrombotic regiment following PCI in patients with long-term indication for chronic oral anticoagulant.

Trial	Years of Publication	Main Inclusion Criteria	Proportion of Patient Presenting with ACS	Antithrombotic Regimen Evaluated	Main Results	Number Needed to Prevent One Ischemic Outcome	Number Needed to Prevent One Bleeding Outcome
WOEST [58]	2013	Indication for Oral Anticoagulation and PCI	155/563 (27.5%)	DAT (Clopidogrel +VKA) vs. TAT (Aspirin + Clopidogrel + VKA)	Reduced Risk of Bleeding with DAT (HR 0.36 95% CI 0.26–0.50) And MACE (HR 0.60 95% CI 0.38–0.94)	15	4 (For Any Bleeding) 42 (For TIMI Major Bleeding)
PIONEER AF-PCI [59]	2016	Non-valvular AF and PCI with coronary stent implantation	1096/2124 (51.6%)	DAT With Rivaroxaban (15 mg Once Daily) + P2Y_12_ Inhibitors and TAT with Rivaroxaban (2.5 mg Twice Daily) + Aspirin + Clopidogrel Or VKA + Aspirin + Clopidogrel	DAT Was Associated with Reduced Risk of Clinically Significant Bleeding (HR 0.59 95%CI 0.47–0.76) Vs. TAT with VKA + Aspirin, Without Significant Difference in Term of Ischemic Events	-	10
RE-DUAL PCI [60]	2017	Non valvular AF Successful PCI < 120 h	2007/2725 (73.7%)	TAT With Dabigatran (110 Mg Twice Daily Or 150 mg Twice Daily) + P2Y_12_ Inhibitors Vs. TAT with VKA + Aspirin+ P2Y_12_ Inhibitors	Both Regimens of DAT Were Associated with Reduced Risk of ISTH Major or Clinically Relevant Bleeding (110 mg B.I.D. HR 0.52 95%CI 0.42–0.63 And 150 mg B.I.D. HR 0.72 95%CI0.58–0.88)	-	9 For Dabigatran 110 mg Twice Daily And 18 For 150 mg Twice Daily
AUGUSTUS [61]	2019	AF and recent PCI or ACS with planned used of at least 6 months of P2Y_12_	2811/4614 (60.9%)	TAT With Apixaban or VKA Vs. DAT With Apixaban Or VKA +Aspirin+P2Y_12_ Inhibitors	DAT Was Associated with Reduced Risk of ISTH Major or Clinically Relevant Bleeding (HR 0.53 95%CI 0.45–0.63) Without Significant Difference In Term Of Ischemic Events	-	14
ENTRUST-AF PCI [62]	2019	Non valvular AF and PCI procedure for stable CAD or ACS	777/1506 (51.6%)	DAT With Edoxaban 60 Mg Twice Daily +P2Y_12_ Inhibitor or TAT With VKA+Aspirin+P2Y_12_ Inhibitors	DAT Was Not Significantly Associated with Reduced Risk of ISTH Major Or Clinically Relevant Bleeding (HR 0.83 95%CI 0.65–1.05) Without Significant Difference For Ischemic Events	-	-

DAT: dual antithrombotic therapy; TAT: Triple antithrombotic therapy; VKA: vitamin K antagonist; ISTH International Society of Thrombosis and haemostasis; AF: atrial fibrillation; PCI percutaneous coronary intervention; TIMI: Thrombolysis in Myocardial Infarction.
